# Efficacy of DAP coated with bacterial strains and their metabolites for soil phosphorus availability and maize growth

**DOI:** 10.1038/s41598-024-61817-6

**Published:** 2024-05-18

**Authors:** Sadia Murad, Maqshoof Ahmad, Azhar Hussain, Sajjad Ali, Nadhir Al-Ansari, Mohamed A. Mattar

**Affiliations:** 1 Department of Soil Science, Faculty of Agriculture and Environment, The Islamia University of Bahawalpur, Bahawalpur, 63100 Pakistan; 2 Department of Entomology, Faculty of Agriculture and Environment, The Islamia University of Bahawalpur, Bahawalpur, 63100 Pakistan; 3 Department of Civil, Environmental and Natural Resources Engineering, Lulea University of Technology, 97187 Lulea, Sweden; 4 Department of Agricultural Engineering, College of Food and Agriculture Sciences, King Saud University, P.O. Box 2460, Riyadh, 11451 Saudi Arabia

**Keywords:** Coated DAP, *Bacillus* spp., Metabolites, ALP gene, P solubilization, P release pattern, Agroecology, Plant sciences

## Abstract

Phosphorus (P) use efficiency in alkaline/calcareous soils is only 20% due to precipitation of P_2_O_5_ with calcium and magnesium. However, coating Diammonium Phosphate (DAP) with phosphorus solubilizing bacteria (PSB) is more appropriate to increase fertilizer use efficiency. Therefore, with the aim to use inorganic fertilizers more effectively present study was conducted to investigate comparative effect of coated DAP with PSB strains *Bacillus subtilis* ZE15 (MN003400), *Bacillus subtilis* ZR3 (MN007185), *Bacillus megaterium* ZE32 (MN003401) and *Bacillus megaterium* ZR19 (MN007186) and their extracted metabolites with uncoated DAP under axenic conditions. Gene sequencing was done against various sources of phosphorus to analyze genes responsible for phosphatase activity. Alkaline phosphatase (ALP) gene amplicon of 380bp from all tested strains was showed in 1% w/v gel. Release pattern of P was also improved with coated fertilizer. The results showed that coated phosphatic fertilizer enhanced shoot dry weight by 43 and 46% under bacterial and metabolites coating respectively. Shoot and root length up to 44 and 42% with metabolites coated DAP and 41% with bacterial coated DAP. Physiological attributes also showed significant improvement with coated DAP over conventional. The results supported the application of coated DAP as a useful medium to raise crop yield even at lower application rates i.e., 50 and 75% DAP than non-coated 100% DAP application which advocated this coating technique a promising approach for advancing circular economy and sustainable development in modern agriculture.

## Introduction

Phosphorus in calcareous soils becomes fixed with Ca^+2^ and Mg^+2^ and in acidic soils mainly with Al^+3^ oxides which results in limited availability of this vital macronutrient for growth and development of crops in many agroecosystems^1^. P fertilizers are applied in large quantities to maintain soil fertility and to improve crop yield^2^. Deficiency of P reduces major metabolic processes during plant growth like respiration and photosynthesis^3^. Worldwide, crop growth is limited by 40% due to P deficiency^4^. However, precipitation, sorption, immobilization, and the PUE is just 10–15% in different crops^5^. Effective management of P fertilizers is important to increase PUE^6,7^. Different ecofriendly approaches for improving P availability have been suggested, such as application of P-solubilizing bacteria^8^, combined application of P fertilizer and biochar^9^, applying P fertilizers as foliar application^10^. The ability of P-solubilizing microorganisms is considered as one of the significant characteristics to overcome plants’ requirement for phosphate nutrition. The mechanisms by which PSB converts phosphate into soluble forms involves the secretion of organic acids with low molecular weight such as citric, oxalic, and succinic acids, whose carboxyl and hydroxyl groups chelate the cations linked to phosphate^11^, synthesis of phosphatase enzymes such as phytases and phosphatases, oxidation–reduction reactions^12^ and ion chelators such as siderophores that facilitate the easy uptake of phosphorus by plants^13^. Coated fertilizers are widely used to improve fertilizer use efficiency. The use of coated P fertilizers has high PUE in both alkaline and acidic soils as compared to conventional fertilizer due to slow release of P^14^. Coated DAP had a longer P-release duration in pot experiment based on soil accessible P during maize growth^15^. Coated-DAP (CDAP) is slow-release fertilizer that prolonged the P availability for plant uptake; thus, with increasing PUE and crop production it also decreases the environmental pollution caused due to the extensive use of synthetic fertilizers^16,17^. Chen et al.^18^ proved that CDAP in comparison of DAP showed significant increase in maize production and PUE by 9.65 and 7.72%, respectively.

The coating acts as a barrier, preventing the contact of soil-fertilizer and limiting interactions among the nutrients and soil (e.g., P fixation). Coating of P fertilizer with bacterial culture and their extracted metabolites increase its bioavailability as slow-release fertilizer in soil^19^. The active component of *Paecilomyces variotii,* a secondary microbial metabolite extracted from endophytic bacterial strain *P. variotii’s* has potential to control crop genes like small auxin-upregulated gene to enhance the level of auxin in the root tip^20^. This metabolite also increases the uptake of N and P^21^, along with crop growth and yields with better PUE^22^ and could be used as coating material. Recently, a rapid increase in the publications on advantages of slow-release fertilizers by utilizing polymeric material has been made^23,24^. In soil organic phosphorus is released by the activity of microbial phosphatases enzyme^25^. Although plant growth promoting bacteria are used as bio-fertilizers all around the world to enhance plant growth and fertilizer use efficiency^26^. There is little information about their use and development of bacterially impregnated fertilizers, which could be a totally new concept and advanced approach to improve fertilizer use efficiency.

Maize is considered as one of the important food crops and an essential forage^27^, which accounts for almost 1/3 of grain production globally^28^. It plays a significant role for consumption of 2/3 of energy by human’s and provide dietary protein (4.50–9.87%), fat (2.17–4.43%), crude fiber (2.10–3%) carbohydrates contents (44.6–69.6%) and ash (1.10–2.95%)^29^. In Pakistan maize production per unit area is still lower than that of its neighbors. Because a large amount of the applied inorganic fertilizer becomes unavailable, this results in a low PUE (25%). Therefore, it is necessary to improve the efficiency and protection of P fertilizers to maximize production potential of maize cultivar.

The term bacterially impregnation of fertilizer refers to chemical fertilizers that have been coated with PGPB to increase the population of useful microorganisms in the rhizosphere and improve the efficacy of used fertilizers. There has been literature available on the use of microorganisms as seed inoculation to increase the use efficiency of phosphorus with lower application rates^30^, but there is need to investigate the use of microbes as coating material for P fertilizers to improve P release pattern in soil and to minimize P losses. This study has been planned to introduce a novel approach to improve P uptake to increase maize productivity under alkaline conditions and to understand the phosphate solubilization mechanism of *Bacillus subtilis* (ZE15 and ZR3) and *Bacillus megaterium* (ZE32 and ZR19) at molecular level and phosphorus release pattern of DAP in water.

## Materials and methods

The experiment was carried out at the Soil Microbiology and Biotechnology laboratory of Department of Soil Science, Faculty of Agriculture and Environment, The Islamia University of Bahawalpur. Pre-identified rhizobacterial strains; *Bacillus subtilis* ZE15 (MN003400), *Bacillus subtilis* ZR3 (MN007185), *Bacillus megaterium* ZE32 (MN003401) and *Bacillus megaterium* ZR19 (MN007186) by Iqbal et al.^31^ were used for the identification of ALP genes and DAP impregnation was done with bacterial isolates and metabolites extracted from these strains in order to obtain maximum yield of maize crop and to improve use efficiency of phosphate fertilizers in alkaline/calcareous soils so that inorganic fertilizer will be efficiently used.

### ALP gene identification against various source of phosphorus

According to the procedure of Mahuku^32^ DNA from overnight bacterial culture was isolated. According to Sakurai et al.^33^, ALPS-F730 (50-CAGTGGGACGACCACGAGGT-30) and ALPS-R1101 (50-GAGGCCGATCGGC-ATGTCG-30) primers were used for the identification of ALP gene and amplification of gene was carried out in thermo cycler (Labcycler, Sensoquest GmbH, Germany). Agarose gel (1% w/v) in TAE buffer was used to resolve PCR product 120 Volts for 45 min. Image of the gel was captured through quantum gel system and editing was done by using MS-PowerPoint version 2010 and the quality of the figures was improved using photoshop-2023.

### DAP coating with bacterial culture and metabolites

Diammonium phosphate fertilizer was purchased from Fauji Fertilizer Company (FFC), Pakistan. The granules were sieved to select homogenous granules with 2–4 mm diameter. After 72 h of incubation in shaking incubator bacterial culture and their extracted metabolites were used as coating material by spraying with the help of spray bottle on the surface of DAP granules. The process was repeated 5 times on the coated DAP. After final coating the DAP fertilizer was stored at room temperature in polythene bags.

#### Metabolites extraction from bacterial culture

Following the protocol of Politz et al.^34^, metabolites was extracted from bacterial culture by extraction and then methylation. According to this method disinfected Pickovskaya broth culture (19.9 mL) was taken in 50 mL Eppendorf tubes and was immunized with 0.1 mL bacterial culture having 1.0 optical density (OD) at 600 nm. After that, the tubes were incubated at 30 ± 1 °C for 10 days at 125 rpm. At the stationary point 3 mL from each tube was taken in separate glass centrifuge tube and 0.1 mL of glacial acetic acid and 5 mL of 1:1 chloroform methanol mixture was added in tubes. Each tube was vortex and centrifuged for 10 min at 1000×*g*. To separate the lower aqueous layer separation funnel was used and nitrogen steam was given to evaporate chloroform remaining dried residues. One milliliter of 1% sulfuric methanol solution was added to the same tube for methylation and sample was heated at 80 °C for 1 h. The sample was cooled on low temperature (in ice) and 2 mL *n*-hexane. After that, 1 mL of saturated NaCl and 2 mL of sterile distilled water was added. After gentle shaking the upper organic layer was separated.

#### Phosphorus release behavior of coated DAP in water

Uncoated, bacterial and metabolite coated DAP (10 g) was taken separately in glass beakers at room temperature and volume was made up to 100 mL by distilled water with continuously gentle stirring to get homogenous system. The 0.1 mL of solution was taken and diluted after different time intervals and absorbance was noted on spectrophotometer for P determination. The concentration of phosphorus was determined on spectrophotometer at 480 nm wavelength using the protocol described by AFNOR T90-022 (Association française de normalisation, 1983).

### Phosphorus solubilization through synthetic organic acids

Bacterial isolates and synthetic organic acids form Halo zone through organic acid production. Phosphorus solubilization through organic acids production by microbes was confirmed by relating Halo zone diameter formed by bacterial cultures and synthetic organic acids. Stock solutions of different organic acids including acetic acid, butyric acid and propionic acid was prepared in double distilled sterilized water (ddH_2_O). The solution of organic acids having different concentrations was prepared separately. One day old bacterial culture was vaccinated on phosphorus amended salt media. Three holes in media were made first for strain, second for specific organic acid, while the third for mixture of organic acids. Eighty-five microliters (85 µL) of each were injected in Agar holes and plates were incubated at 25 °C for 72 h to measure halo zone diameter. Phosphate solubilization efficiency (SE) and solubilization index (SI) were measured by using the formulas given by Qureshi et al.^35^.

1 $$\mathrm{SE }(\mathrm{\%}) = \frac{Halo\, zone\, diamter}{Colony \,diamter} \times 100,$$

2 $${\text{SI}}=\frac{Halo \,zone\, diamter+colony\, diamter}{Colony\, diamter}.$$

### Jar trail

The jar trial was conducted at Soil Microbiology and Biotechnology Laboratory, Department of Soil Science. Sterilized and autoclaved sand (600 g) was filled in jars having diameter and height of 4.5ʹʹ × 4.5ʹʹ. Uncoated and coated DAP fertilizer was applied before sowing of maize (4 seeds pot^−1^) according to the treatment plan as described in Table 1. Each treatment was replicated 3 times. Maize crop was sown under 60% of relative humidity, 10–12 h of ambient light and at ~ 28 °C temperature. After 26 days the crop was harvested, and different morphological and physiological parameters were analyzed.

**Table 1:** Self-explanation of treatments (% of DAP fertilizer applied).

Treatments	Un-inoculated/bacterial inoculated/metabolites inoculated
Control	Without DAP
25%	0.045 g DAP pot^−1^
50%	0.089 g DAP pot^−1^
75%	0.135 g DAP pot^−1^
100%	0.179 g DAP pot^−1^

#### Growth parameters

Morphological parameters such as shoot, and root fresh weight were measured by using electrical balance and root and shoot lengths were taken by measuring tape. After that, samples were air dried and stored in a dry cool place for further analysis.

#### Leaf area

Fully expended fresh leaf was taken from each plant and leaf area was determined using leaf scanner (Win FOLIA Pro, STD, 2016, Netherland).

#### Post-harvest sand analysis

After crop harvesting, sand samples from maize rhizosphere were collected and analyzed for different physico-chemical properties. Composite sand samples were air dried and sieved through a 2 mm mesh size sieve. Samples were stored in refrigerator at 4 °C temperature and were analyzed within seven days. The serial dilution and pour plate method was used to calculate bacterial population as colony forming unit (CFU)^36^. Organic matter was measured using the method described by Nelson and Sommers^37^. The method of Watanabe and Olsen^38^, was used to analyze available phosphorus using UV–Visible spectrophotometer. Chloroform fumigation and extraction method^39,40^ was used to determine microbial biomass carbon and nitrogen.

### Statistical analysis

Statistical analysis was performed using factorial design by two-way ANOVA interaction using Statistix 8.1 software and means were compared using Tukey’s HSD at p ≤ 0.05^41^.

### Statement

The study was conducted in accordance with relevant guidelines and legislation is also acceptable.

## Results

### Identification of alkaline phosphatase (ALP) gene

**Figure 1:**
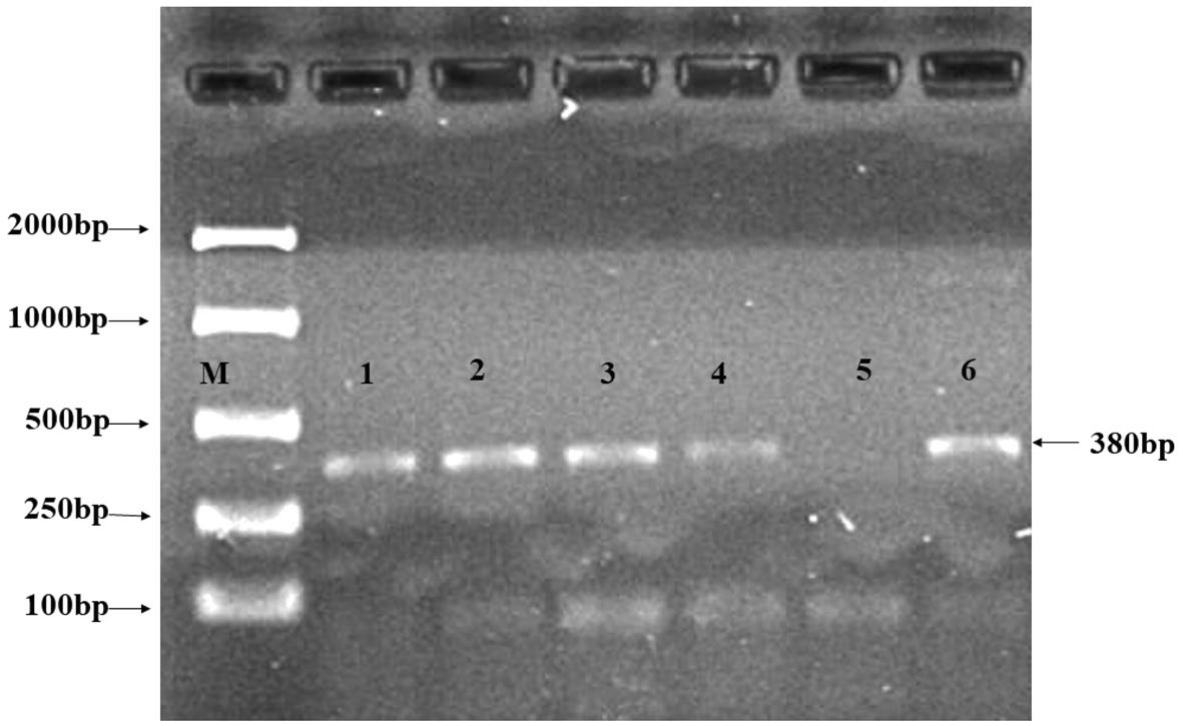
Bacilli species showing alkaline phosphatase gene amplicon of 380bp in polymerase chain reaction. Molecular weight marker is represented by M, *Bacillus subtilis* ZE15 by 1*, Bacillus megaterium* ZE32 by 2*, Bacillus subtilis* ZR3 by 3 and *Bacillus megaterium* ZR19 by 4. While negative control without template was represented by 5 and positive control by 6.

DNA from all selected bacterial isolates was extracted, used in polymerase chain reaction (PCR) and subjected to gel separation (Supplementary Fig. 1). Results showed that all the tested strains showed amplicon of 380bp against alkaline phosphatase (ALP) gene in 1% w/v agarose gel (Fig. 1) In rhizosphere alkaline phosphatase activity is due to presence of ALP gene, which improves root growth and release of organic acids in the root exudates. The presence of ALP gene in tested strains indicates their ability to solubilize phosphorus through increased enzyme activity and organic acids secretion in the rhizosphere.

### Phosphorus release behavior of coated DAP in water

**Figure 2:**
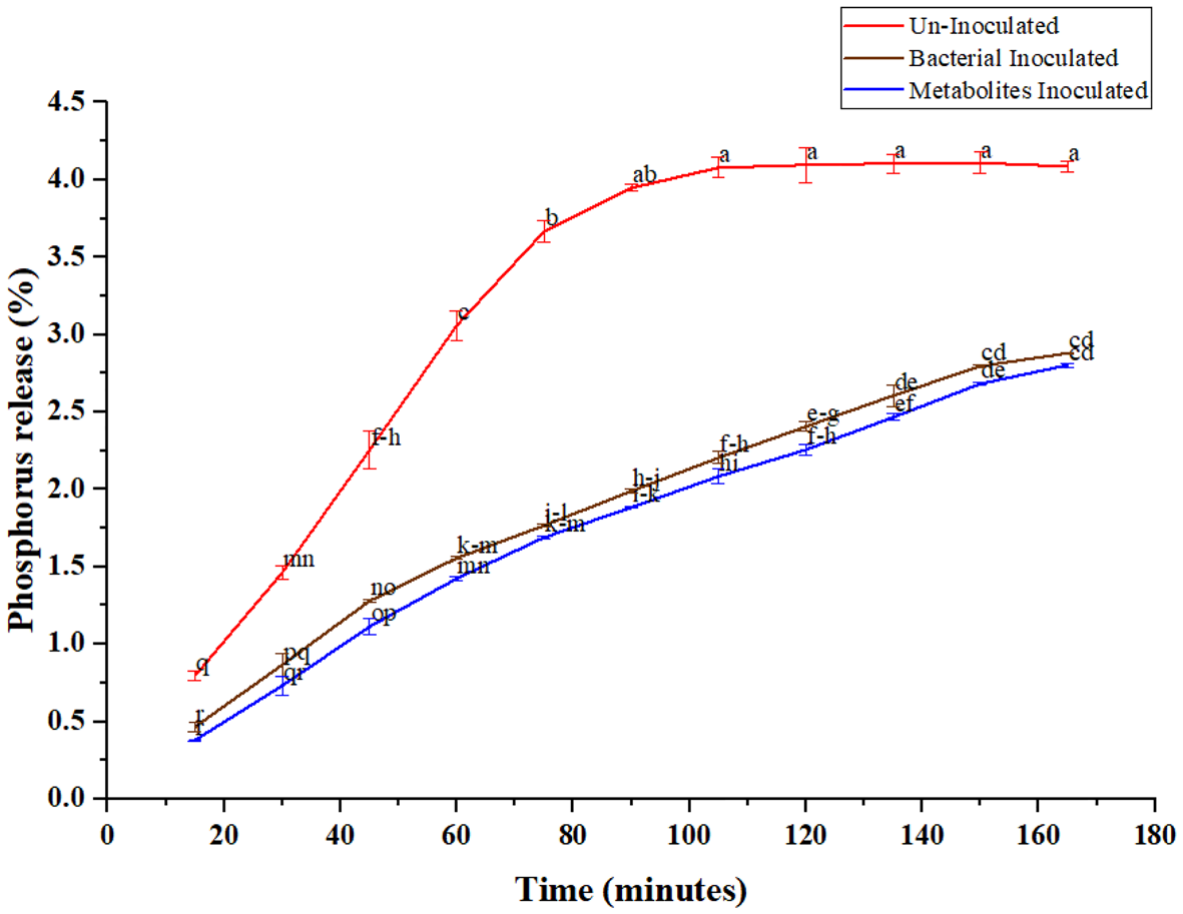
Phosphorus release rate in distilled water from Un-Inoculated, bacterial inoculated and metabolites inoculated DAP at room temperature from different time intervals. Values with similar latter (s) are statistically non-significant to each other at p ≤ 0.05.

The release pattern of coated DAP with selected strains and their extracted metabolites was measured in comparison to control (uncoated DAP) to assess their application in agriculture. The release percentages of phosphorus from coated and uncoated DAP in water after different time intervals at 7 pH and at room temperature was shown in Fig. 2. Results showed that almost all phosphorus released in case of control (uncoated DAP) within less than 2 h. After 105 min, the release pattern of uncoated DAP reached equilibrium stage (complete release). Phosphorus release is much slower from granules of inoculated DAP as compared to un-inoculated and gain equilibrium after 170 min both from bacterial coated and metabolites inoculated DAP. Phosphorus was gradually available to plants for long time when DAP was coated with bacterial culture and their extracted metabolites. Maximum phosphorus concentration was available after 150 and 165 min from bacterial and metabolites coating, respectively, as compared to control indicating significant slow-release properties of phosphorus.

### Phosphorus solubilization through synthetic organic acids

**Figure 3:**
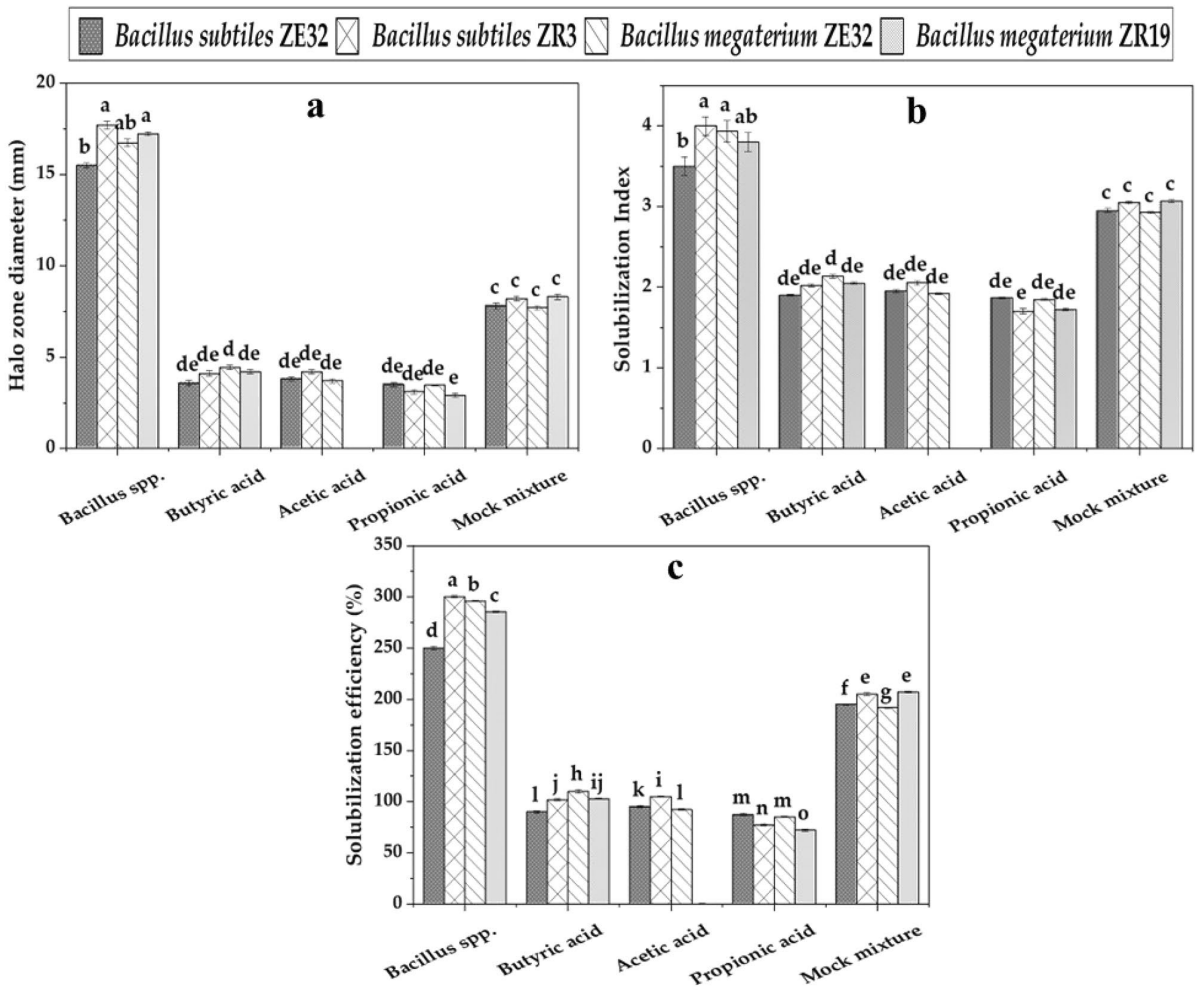
Phosphorus solubilization through selected *Bacillus subtilis* and *Bacillus megaterium* species and synthetic organic acids. Means with different alphabets differ statistically from each other (p ≤ 0.05).

To compare p solubilization efficiency of different organic acids and their mock mixtures with bacterial strains were tested in an in vitro experiment. For comparison, ZE15, ZR3, ZE32, ZR19, and synthetic organic acid (propionic, butyric, and acetic acid) were inoculated in media (Fig. 3). Maximum solubilization was shown by *Bacillus subtilis* ZR3 with 17.7 mm halo zone diameter as shown in Fig. 3a, solubilization index was 4 (Fig. 3b), and solubilization efficiency was 300% (Fig. 3c). Next better P solubilizing isolate was *Bacillus megaterium* ZR19 with 17.2 mm halo zone diameter, 285% SE and 3.9 SI. Among tested synthetic organic acids, acetic acid showed maximum P solubilization as its concentration was detected from *Bacillus subtilis* (ZE15, ZR3) showed halo zone formation (3.8mm), SE (95%), SI (1.9) and (4.2 mm), SE (105%), SI (2.05) respectively while it remains unable to produche halo zone among ZR19 isolate. While among *Bacillus megaterium* (ZE32, ZR19) butyric acid showed maximum P solubilization with 4.4 mm halo zone diameter, 110% SE, 2.1 SI and 4.2 mm diameter, 103% SE and 2.05 SI respectively. Application of mock mixture ZE15 showed halo zone formation having diameter 7.8 mm, SE 195% and SI 2.95. Mock mixture of ZR19 showed halo zone diameter (8.3), SE (207%) and SI (3.07). Formation of halo zone through butyric acid also showed P solubilization with maximum 3.5 mm diameter, 90% SE and 1.9 SI from ZE15 isolate and minimum 4.1 mm diameter, 102% SE and 2.02 SI from ZR3**.**

### Effectiveness of bacterial and metabolites inoculated DAP on growth characteristics of maize

Coating of DAP fertilizer showed improvement in Shoot dry weight as compared to conventional DAP which was without coating. Data shown in Table 2 revealed that at 25%, 50%, 75%, and 100% of recommended DAP fertilizer, shoot dry weight was increased by 14, 25, 31, and 43% respectively in case of bacterial inoculated and 18, 23, 31 and 46% under metabolites inoculated as compared to control which was without DAP. Un-inoculated fertilizer application at full dose showed only 26% increase in shoot dry weight as compared to control. The coated fertilizer also showed significant results related to shoot length and root length of maize crop under different doses of applied fertilizer. Similarly root dry weight was also significantly increased due to application of inoculated DAP. Significant results were observed when 75% and 100% of recommended dose was applied with 35 and 44% increase in root dry biomass with bacterial inoculated fertilizer and 41 and 45% with metabolites inoculated fertilizer. Coated DAP even at 50% and 75% application rate showed better results as compared to 100% application of conventional fertilizer.

**Table 2:** Effectiveness of bacterial and metabolites inoculated DAP on growth characteristics of maize.

Treatments	Shoot length (cm)			Root length (cm)		
	Un-inoculated	Bacterial inoculated	Metabolites inoculated	Un-inoculated	Bacterial inoculated	Metabolites inoculated
Control	29 ± 0.57^g^	29 ± 1.15^fg^	30 ± 0.57^fg^	11 ± 0.57^e^	11 ± 0.57^e^	11 ± 0.50^e^
25%	33 ± 1.52^ef^	35 ± 2.08^de^	36 ± 1.52^c–e^	12 ± 1.00^de^	13 ± 0.57^b–e^	13 ± 1.32^a–e^
50%	36 ± 1.00^c–e^	38 ± 2.08^b–d^	40 ± 2.00^a–c^	12 ± 2.00^c–e^	14 ± 0.28^a–d^	14 ± 1.04^a–e^
75%	36 ± 1.00^c–e^	42 ± 1.00^ab^	42 ± 2.00^ab^	13 ± 1.04^b–e^	15 ± 0.50^a–c^	16 ± 0.57^ab^
100%	36 ± 1.15^c–e^	42 ± 1.00^ab^	43 ± 0.57^a^	14 ± 1.00^a–e^	16 ± 1.00^ab^	16 ± 0.57^a^
HSD(*p* ≤ 0.05)	0.040			0.032		

Treatments	Shoot dry weight (g)			Root dry weight (g)		
	Un-inoculated	Bacterial inoculated	Metabolites inoculated	Un-inoculated	Bacterial inoculated	Metabolites inoculated
Control	0.15 ± 0.01^f^	0.16 ± 0.01^ef^	0.16 ± 0.01^ef^	0.10 ± 0.00^d^	0.11 ± 0.00^d^	0.11 ± 0.00^d^
25%	0.16 ± 0.00^d–f^	0.18 ± 0.01^c–f^	0.19 ± 0.01^b–f^	0.12 ± 0.01^cd^	0.12 ± 0.01^cd^	0.13 ± 0.01^a–d^
50%	0.18 ± 0.01^c–f^	0.20 ± 0.01^a–e^	0.19 ± 0.00^a–e^	0.12 ± 0.00^cd^	0.13 ± 0.00^a–d^	0.13 ± 0.00^a–d^
75%	0.18 ± 0.02^c–f^	0.20 ± 0.01^a–d^	0.21 ± 0.01^a–c^	0.12 ± 0.01^b–d^	0.15 ± 0.01^a–c^	0.15 ± 0.01^ab^
100%	0.19 ± 0.01^b–f^	0.23 ± 0.01^ab^	0.23 ± 0.01^a^	0.14 ± 0.01^a–d^	0.16 ± 0.01^a^	0.16 ± 0.01^a^
HSD(*p* ≤ 0.05)	0.040			0.032		

Data in the table is shown as means of three replicates ± standard error. Values with same latter (s) within a column are statistically non-significant to each other at p ≤ 0.05.

### Effectiveness of bacterial and metabolites inoculated DAP on leaf area of maize

**Figure 4:**
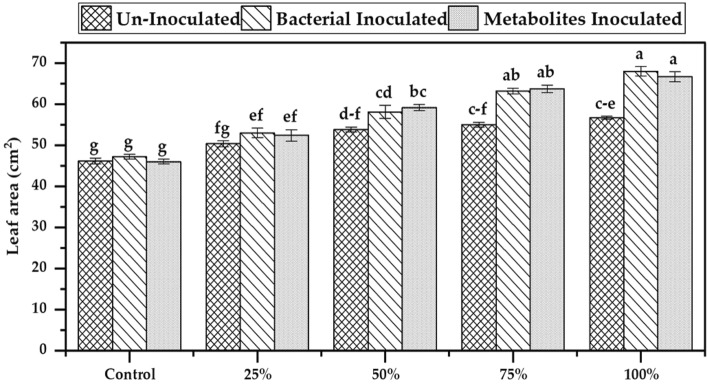
Effectiveness of bacterial and metabolites inoculated DAP on Leaf area of maize crop in jar trail. Bars with different alphabets differ statistically from each other (p ≤ 0.05).

Bacterial strains and their extracted metabolites showed significant improvements in crop physiological characteristics such as leaf area when they were applied as coating material for DAP fertilizer. Results related to leaf area showed 38% and 40% increase at 100% required dose of bacterial and metabolites inoculated DAP respectively as compared to their respective control (Fig. 4). While similar dose of un-inoculated DAP showed 21% increase in leaf area which is almost 17 and 19% lower than the improvement observed from bacterial, and metabolites coated DAP application. Coating improved leaf area DAP. Lowest increase in leaf area was observed in control conditions where no DAP was applied under all conditions.

### Effectiveness of bacterial and metabolites inoculated DAP on physicochemical and biological properties of sand

**Figure 5:**
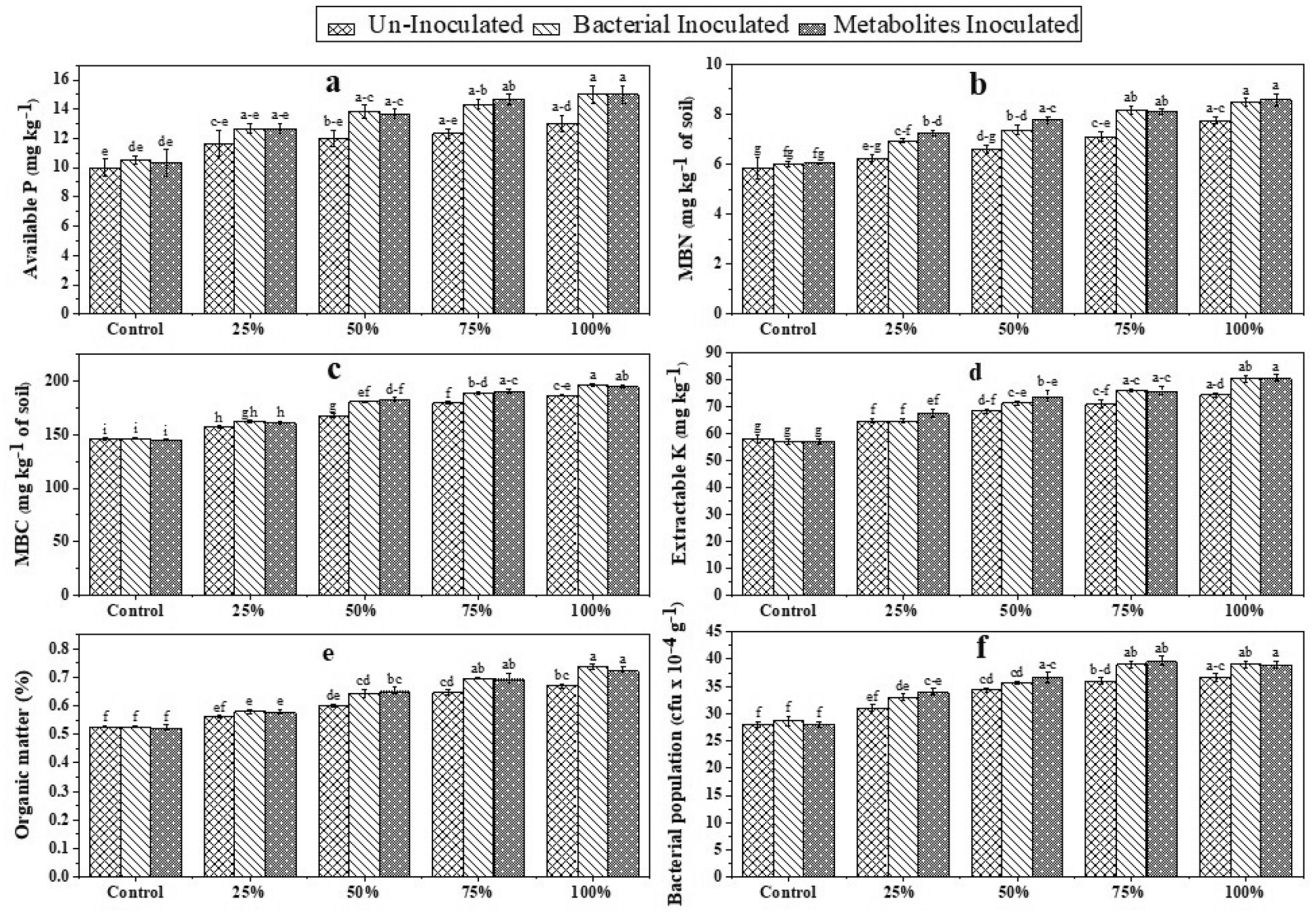
Effectiveness of bacterial and metabolites inoculated DAP on available P (**a**), MBN (**b**), MBC (**c**), extractable K (**d**), organic matter (**e**) and bacterial population of post-harvest rhizospheric sand (**f**). Bars with different alphabets differ statistically from each other (p ≤ 0.05).

The effectiveness of bacterial and metabolites coated DAP on different rhizospheric sand properties in jar trail were described in Fig. 5. Statistical analysis revealed that bacterial and metabolites inoculated DAP showed statistical similar impact on available phosphorous concentration whereas maximum 42 and 45% increase was observed respectively when 100% required DAP was applied. Un-Inoculated DAP at 100% required dose showed 30% increase in available phosphorus as compared to its control which was without DAP application (Fig. 5a). Microbial biomass carbon (MBC) and microbial biomass nitrogen (MBN) also showed significant results when jars were applied with inoculated DAP as compared to un-inoculated (Fig. 5b,c). Bacterial and metabolites inoculated DAP increased MBC by 35% and 37% respectively at 100% dose of DAP while MBN was improved by 41% under same rate of application as compared to control. Bacterial and metabolites inoculated DAP showed maximum increase in extractable potassium concentration in rhizospheric sand sample (Fig. 5d) i.e., 40 and 41% respectively as compared to their control when full dose of required DAP was applied followed by 33% increase in extractable potassium when 75% of required bacterial and metabolites inoculated DAP was applied. Similarly, organic matter contents in rhizospheric sand of jar trail were improved by the application of inoculated DAP as compared to un-inoculated. An increase of 39 and 38% in organic matter was observed by 100% dose of bacterial and metabolites inoculated DAP respectively. While the same dose of un-inoculated DAP application showed only 27% improvement in organic matter as compared to control (Fig. 5e). The next better results were observed at 75% required dose of bacterial and metabolites inoculated DAP with 32 and 33% improvement. As compared to un-inoculated, inoculated DAP improved bacterial population more significantly. Maximum increase (41%) was observed when 75% of required metabolites inoculated DAP was applied followed by 39% increase obtained by 100% application of metabolites coated DAP. While 36% increase was observed by 75 and 100% dose of bacterial coated DAP as compared to control (Fig. 5f).

## Discussion

More than 90% of Pakistani soils are characterized as calcareous sandy soils which consists of high calcium carbonate contents, and alkaline pH (> 8) with low nutrient availability and less than 1% organic matter^42^. As P is second most important macronutrient for plant growth and its low application along with all above factors results in incredibly low crop yield and productivity in Pakistan. Therefore, for high yield of maize crop it is necessary to make balance between fertilizer application and crop output^43^. The use of phosphate solubilizing bacteria could maintain the balance between nutrient uptake and crop output. Excretion of organic acids, extracellular enzymes’ production, and secretion of siderophores are the main mechanisms performed by phosphorus solubilizing microbes for improving soil fertility status and uptake of available P by plants^44^. Structure and functions of microbial community in soil and plant microbe’s interaction is directly affected by metabolites released by PSMs, which will stimulate physiological changes in plants and thus accelerating growth of plant^45^. In this study, we analyzed the potential of *Bacillus subtilis* and *Bacillus megaterium* for improving growth and nutrient uptake by plants. Results from present study revealed that DAP coated with PSB and their extracted metabolites increased growth attributes of maize cultivar including shoot and root biomass. According to Ref.^46^, dual benefits of coated DAP is responsible for improving growth and yield based parameters in wheat through different growth promoting mechanisms.

Four strains based on their performance were tested for ALP gene identification at different levels of soluble phosphorus. All the tested isolates showed amplification for ALP gene. Phosphate solubilization was mainly due to the presence of ALP gene and organic acids. In literature, it is mentioned that alkaline phosphatase activity was due to the release of organic acids and added organic matter. Addition of organic matter increased the alkaline phosphatase activity^47^. The P release rate of the coated granules reached the equilibrium stage approximately after 170 min as compared to uncoated DAP granules (control), which were totally solubilized within less than 2 h. Phosphorus may release from coated DAP according to the nutritional requirement of plant^48^. Holmen et al.^49^ stated that the coating prevents P from being fixed and rapid dissolution of DAP and losing of P through subsurface flow and surface runoff making it available for longer period. Slow release of P from coated DAP may also be due to the thickness of coated layer which was made by 5–6 subsequent coatings on initial coating. In another study, it was stated that due to high thickness of coated material a resistance pathway was formed which slowed down the release pattern of P^50^. Indeed, Da Cruz et al.^16^, reported that the significant delays in phosphorus release was observed when DAP was coated with castor PU coatings (3.0 and 4.5 wt%). For 3.0 wt% release of P detected was 80% in 50 h and for 4.5 wt% same release rate was observed in 75 h. Our results indicated that nutrient availability can be increased for longer time with coated fertilizer application.

Coating DAP with bacterial isolates and their extracted metabolites were found to improve sand physicochemical and biological characteristics for maize growth. The results of our study showed that application of coated fertilizer not only increase maize yield but also improve sand physicochemical and biological properties. The results are in accord with Wang et al.^22^, who reported that *Paecilomyces variotii* extract and coated urea fertilizer not only improve plant physiological functions but also increase soil physicochemical and biological attributes. Findings of this study showed that application of coated DAP enhanced plant biomass and physiological characters. Our results were confirmed by findings of Beltran-Medina et al.^51^, that physiological attributes of plants were improved due to the application of PSB. The application of PSB reduces the pH by releasing organic acids in the rhizosphere, which increases the availability of inorganic P for plants. Our results regarding increased P availability are corelated with Ma et al.^52^, who investigated that coating of DAP increased the available P to maize crop. The application of coated DAP also improved organic matter contents in the growth medium (sand) which may be due to vigorous plant growth due to better P availability which leads to an increase in root biomass and root exudates creating active sand microbiome interaction that contribute to organic matter pool. Consequently, coated phosphatic fertilizers were more economical compared to conventional fertilizers even at reduced application rate.

## Conclusion

In conclusion, coating of DAP fertilizer with phosphorus solubilizing bacteria and metabolites could be effective as compared to conventional fertilizer to get continuous and slow release of P for longer time interval. Results of this study showed that coating of mineral fertilizer is very effective in increasing growth and PUE in maize even at lower rates of application i.e., at 50 and 75% as compared to full dose of conventional fertilizer. However, as a perspective, there is need to assess the potential of such PGPB-impregnated fertilizers to enhance the growth and yield of agricultural crops under field conditions.

### Supplementary Information

Supplementary Figure 1.

## Data Availability

The datasets used and/or analyzed during the current study are available from the corresponding author on reasonable request.
