# Supplementary figures and images for: Efficacy of DAP coated with bacterial strains and their metabolites for soil phosphorus availability and maize growth
Source: Sci Rep. 2024 May 18;14:11389. doi: 10.1038/s41598-024-61817-6 (PMC11102545; doi:10.1038/s41598-024-61817-6)

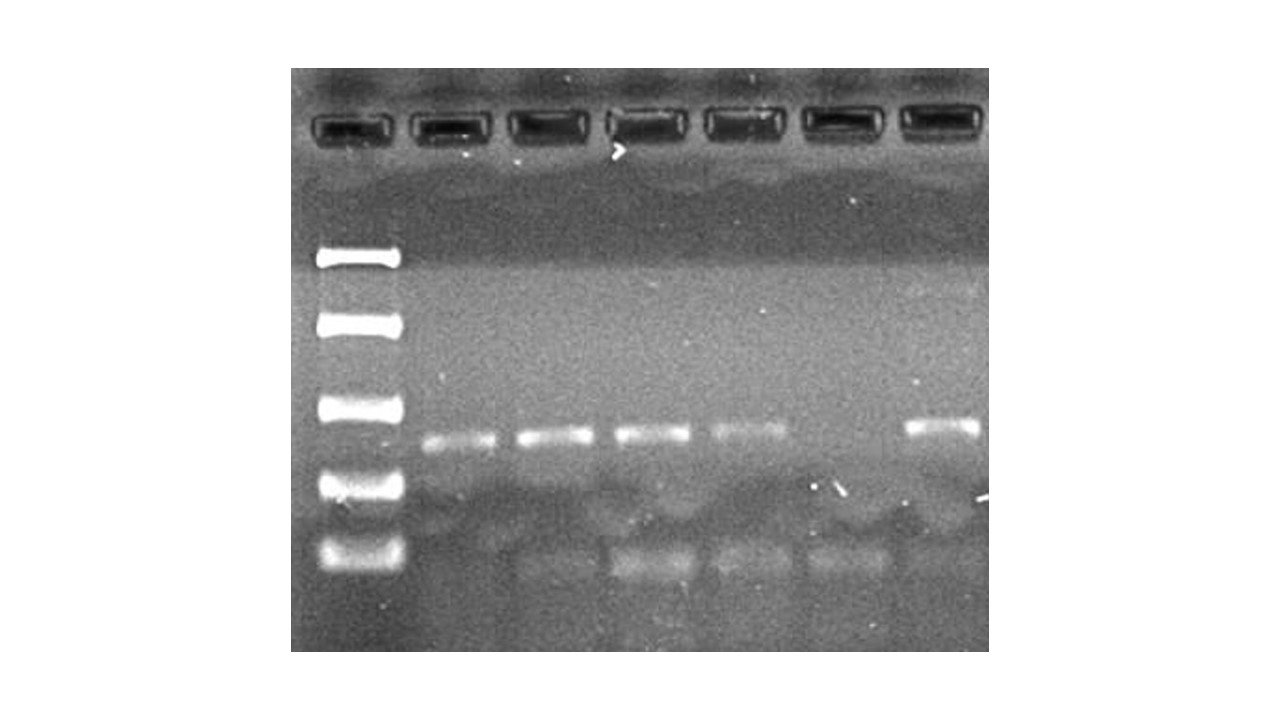

Supplement: Supplementary file 1 — Supplementary Figure 1. [file 41598_2024_61817_MOESM1_ESM.jpeg]
